# Charles Bonnet Syndrome in the Setting of a Traumatic Brain Injury

**DOI:** 10.7759/cureus.29293

**Published:** 2022-09-18

**Authors:** Ricardo Irizarry, Ariel Sosa Gomez, Jean Tamayo Acosta, Leonel Gonzalez Diaz

**Affiliations:** 1 Psychiatry, South Texas Health System, McAllen, USA; 2 Psychiatry, Tropical Texas Behavioral Health, McAllen, USA; 3 Medicine, Saint James School of Medicine, Miami, USA; 4 Medicine, University of Medicine and Health Sciences, Bayamon, PRI; 5 Family Medicine, Ross University School of Medicine, Miami, USA

**Keywords:** atypical anti-psychotic, selective serotonin reuptake inhibitor (ssri), visual hallucinations, olanzapine, psychosis, alcoholism, escitalopram, topiramate, traumatic brain injury, charles bonnet syndrome

## Abstract

Traumatic brain injuries are often associated with a broad range of neuropsychiatric sequelae, for which no straightforward treatment approach is established. Historically, the treatment of traumatic brain injuries has been tailored towards the symptoms presented by the patient. In this paper, we present the case of a 42-year-old male with a past medical history significant for retinitis pigmentosa who suffered a traumatic brain injury and subsequently developed visual hallucinations consistent with Charles Bonnet syndrome.

## Introduction

Charles Bonnet syndrome (CBS) is a health condition characterized by visual hallucinatory episodes, which commonly manifest in individuals with distorted vision [1]. These episodes may present themselves as a series of events or alternating sequences of images or sometimes can appear as a representation of certain places. It is crucial to comprehend that hallucinations linked to this syndrome are connected to a deteriorating vision and are not connected in any way with abnormalities in a person's mental condition. Subsequently, personal interventions should be implemented to enable people to seek proper medical treatment for any visual defect they may experience before the conditions worsen [2]. 

Patients suffering from CBS are often individuals who have completely lost their vision (both eyes have been distorted from proper vision). Vision helps people perceive their environment and the things that exist within it; it can be difficult for people suffering from visual disorders to process objects effectively. This syndrome has been attributed to sensory deprivation and neurophysiologic disturbances, and deafferentation from sensory deprivation may cause increased excitability and spontaneous neural activity in the V1 and V2 regions of the visual cortex [3]. Moreso, it is also a syndrome often seen in elderly patients who have visual impairments. Still, this condition may occur in younger patients who do not have visual impairments and who have damage to the optic pathway or within the brain. Visual hallucinations may be associated with damage to the primary visual cortex and deafferentation of the visual association areas. This syndrome arising following a traumatic brain injury (TBI) is not common. However, it has been previously reported as an incident occurring various weeks after the onset of TBI [3].

CBS was discovered and first described by Charles Bonnet in 1760 [4]. This condition is much more prevalent in older people with diminished vision than in younger people, thus ranging between 0.3% and 30%. Research shows that neuropsychological testing is paramount in the diagnostic workup of CBS. It is also estimated that over 100,000 individuals in the United Kingdom have been diagnosed to experience hallucinations and other complications like CBS [5]. Extensive studies regarding this critical condition have established a viable relationship between the eye and the human brain and a deep understanding of brain functions. The brain functionality is suppressed as soon as a person begins experiencing visual problems; hence this situation is linked to the brain's inability to integrate vision properly. The images stored in the brain after failed vision are therefore presented as hallucination episodes.

Herein we describe the case of a 42-year-old man with a significant past medical history of retinitis pigmentosa and an episode of TBI who developed CBS after the injury. This patient was medically stabilized, returned to his baseline functioning, and followed up outpatient to prevent future relapses. 

## Case presentation

This is the case of a 42-year-old male software engineer with a past medical history of traumatic brain injury, pancreatitis, peripheral neuropathy, high blood pressure, bilateral eye blindness secondary to retinitis pigmentosa, metabolic syndrome, allergies, and alcohol abuse. He was admitted to the hospital for one week due to disorganized behavior, aggression, and hallucinations. His home medications included amlodipine 10 mg every day (qd), atorvastatin 10 mg each night at bedtime (qhs), gabapentin 300mg orally (PO) twice a day (bid), glipizide 5 mg PO qd, metformin 1000 mg PO bid, montelukast 10 mg PO qhs, tadalafil 5 mg PO qd (Table 1). 

**Table 1:** Patient medication list prior to admission

Outpatient medications	Dosage	Route	Frequency
Amlodipine	10 mg	Oral	Once daily
Atorvastatin	10 mg	Oral	Once at night
Gabapentin	300 mg	Oral	Twice daily
Glipizide	5 mg	Oral	Once daily
Metformin	1000 mg	Oral	Twice daily
Montelukast	10 mg	Oral	Once at night
Tadalafil	5 mg	Oral	Once daily

Day one

The patient presented with disorganized speech and an inability to respond to questions or follow commands fully. At the time of admission, the patient was accompanied by his mother. The latter reported that the patient had a past medical history significant for TBI one month prior. It was stated that the patient had sustained this injury due to a fall, secondary to retinitis pigmentosa, diagnosed in 2000. It was also reported that the patient presented with nausea and poor appetite nine days prior to admission; in said instance, he was administered a one-time dose of promethazine 25mg PO at an urgent care facility to address the presenting symptoms. Four days before admission, the patient exhibited visual hallucinations, confusion, tremors in the hands and legs, and aggressive behavior. On the day of admission, the patient was brought to the hospital by the police under section eight. His mother reported that the patient had not slept for three days. She also reported that the patient's last alcoholic drink had been over one month prior. Upon admission, the patient was very impulsive, aggressive, and combative and exhibited inappropriate behaviors such as urinating in the hallways.

On physical examination, vital signs were within normal limits. The patient exhibited poor eye contact, a subpar general appearance, and psychomotor delay. His speech had a decreased rate, volume, and tone. When asked about his current mood, he expressed being "ok"; however, this response was not congruent with his affect, which was flat and dysphoric. The patient's thought process was disorganized, with loose associations. Thought content was positive for delusional construct and paranoia. The patient was noted to be responding to internal stimuli and reported visual hallucinations characterized by the initial appearance of children standing around his household, followed by the later appearance of children running around the same household. These hallucinations had a sporadic onset and lasted only seconds to a few minutes. The patient denied auditory hallucinations and suicidal or homicidal ideations; the remaining review of systems was negative. 

The patient's Mini-Mental State Examination (MMSE) [6] score was 16/30; please note that this score was modified for blind patients by omitting the sections which required vision from the calculations - the maximum score was adjusted and prorated to be comparable with 30. His non-prorated score was 12/22 (omitting the eight points corresponding to sessions that required vision to be evaluated). This score indicated severe cognitive impairment. His Patient Health Questionnaire-9 (PHQ-9) [7] reflected a score of nine, indicating that the patient presented mild signs of depression. His Young Mania Rating Scale (YMRS) [8,9] score was 23, indicating mild mania.

The patient was admitted to the psychiatric ward as a preventative measure, with plans to encourage avoidance of visual stimuli from the environment, and placed on one-to-one observation due to his legally blind status and current presentation; as well as stabilization of his acutely ill condition with olanzapine intramuscular (IM) 1ml (5mg). After olanzapine administration, the patient's acute agitation was diminished; nonetheless, his hallucinations, paranoia, and aggressive state failed to subside. Two hours later, a second dose of olanzapine IM 2ml (10 mg) was administered with slightly improved agitation. 

He was subsequently administered topiramate 150 mg PO bid and escitalopram 5 mg PO qd to address the underlying cause of his presentation. His initial preventive diagnosis was traumatic brain injury (TBI), with suspicion of Charles-Bonnet syndrome (CBS).

Inpatient medications included atorvastatin 10 mg PO qhs, insulin aspart NovoLog subcutaneouslu (sq) 1 unit/0.01mL syringe (intermediate scale), metformin 500mg extended-release (XR) PO bid, valsartan 160mg PO qd, and as needed (PRN) olanzapine 5mg every six hours (q6h) (Table 2).

**Table 2:** Patient medication list status post admission Please note topiramate and escitalopram were added after the preliminary diagnosis XR - extended-release

Inpatient medications	Dosage	Route	Frequency
Atorvastatin	10mg	Oral	Once at night
Insulin aspart NovoLog (sq 1 unit/0.01mL syringe)	2-16 units	Subcutaneous	Before meals and at night
Metformin	500mg XR	Oral	Twice daily
Valsartan	160mg	Oral	Once daily
Olanzapine	5mg	Intramuscular	As needed every six hours
Topiramate	150mg	Oral	Twice daily
Escitalopram	5mg	Oral	Once daily

The initial magnetic resonance imaging (MRI) scan showed no acute brain injury, hemorrhage, skull fracture, or extra-axial collection. There was mild cortical volume loss in both hemispheres. The drug panel (10-drug panel) was negative for psychoactive substances, and the ethanol screening was negative. 

Day two

On day two of admission, the patient reported feeling much better. He showed significant improvement in his acutely ill presentation. On physical examination, his mood was found to be stable, without substantial concerns of irritability, anger, or depression. He reported feeling calm with decreased levels of anxiety. The patient continued to deny psychotic symptoms and suicidal or homicidal ideations. He denied having visual hallucinations. At this point, his current medical management was maintained, with plans to monitor his condition's progression closely.

Day three

On day three of admission, the patient was interviewed in his room in the presence of a staff member. No side effects were reported from medications; he had slept well through the night and maintained one-to-one observation due to his legally blind status. The patient exhibited an excellent appetite and spent most of his time in his room. At this point, the patient inquired when he would be allowed to go home and understood the reasoning to keep him hospitalized for further observation. We continued to monitor the patient's sleep, behavior, and appetite. While also providing reassurance, redirection, and emotional support when appropriate.

Day four

On day four of admission, the patient reported feeling very well at the time. His mood had remained stable throughout the previous days, with no significant concerns for irritability, anger, or depression. The patient continued to deny psychotic symptoms and suicidal or homicidal ideations. He denied having visual hallucinations. His MMSE score was 28/30; note that this score was modified for blind patients by omitting the sections which required vision from the calculations. The maximum score was adjusted and prorated to be comparable with 30. This score indicated no cognitive impairment. His PHQ-9 reflected a score of three, indicating that the patient presented minimal signs of depression and did not require treatment. His YMRS score was two, indicating euthymia. The patient requested to be discharged home, and given that he no longer met the criteria for psychiatric hospitalization, he was discharged with recommendations to continue medications as prescribed during his hospitalization with a psychiatric follow-up appointment within one week of discharge. He was also instructed to return to the hospital upon any exacerbation or recurrence of symptoms and educated on proper sleep hygiene and lifestyle modifications.

**Figure 1:**
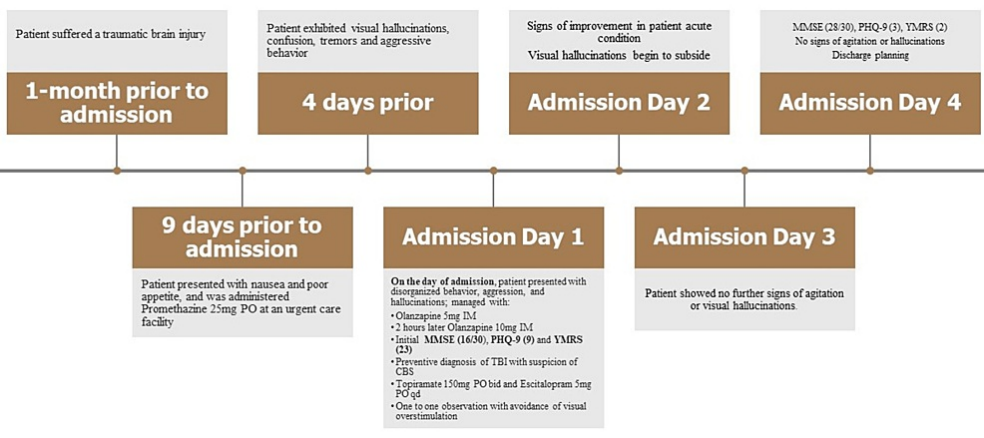
Chronological synopsis of symptoms and management PO -oral; IM - intramuscular; MMSE - Mini-Mental State Examination; PHQ-9 - Patient Health Questionnaire 9; YMRS - Young Mania Rating Scale; TBI - traumatic brain injury; CBS - Charles Bonnet syndrome; bid - twice daily; qd - daily

Figure 1 depicts the chronological synopsis of symptoms and management.

## Discussion

When considering the treatment approach, ruling out differential diagnoses for our patient's acute agitation and altered mental status is paramount to providing appropriate care.

Alcoholic hallucinosis is a rare complication of chronic alcohol abuse characterized by predominantly auditory hallucinations that occur either during or after a period of heavy alcohol consumption [10]. These hallucinations may sometimes be tactile or visual as well. However, unlike in CBS, they are rarely only visual. Previous studies have shown a very low incidence of exclusively visual hallucinations tied to alcoholic hallucinosis, where 3/6493 subjects described having only visual hallucinations during their alcohol withdrawal period [11]. Given our patient's alcohol consumption history, this condition had to be discarded to identify the correct etiology of his visual hallucinations. In addition, his vital signs were within normal limits, with no signs of autonomic dysfunction. The patient's clinical picture in which he presented with disorganized behavior, aggression, and hallucinations for one week, in combination with blood panel results negative for alcohol or other substances of abuse, was enough to rule out the possibility of alcohol withdrawal being the cause of the presenting visual hallucinations.

In the presence of hallucinations and the mild manic state our patient presented with, it was necessary to rule out psychosis secondary to a mood disorder. In this particular case, the patient did not meet the criteria to be diagnosed with depression with psychotic features due to the lack of symptoms corresponding to the current guidelines established by the Diagnostic and Statistical Manual of Mental Disorders, Fifth Edition (DSM-V) [12]. It is unlikely to experience hallucinations when only a mild form of depression is exhibited, primarily when a different etiology can better explain it. In this case, our patient's blindness and TBI explain his current presentation better. 

Traumatic brain injuries must be addressed promptly and treated based on their etiology and presenting symptoms. Topiramate is a medication that may be used to treat various pathologies, including alcohol use disorder and TBI [13]. Given our patient's presentation, this medication was appropriate for initially managing the presumptive underlying condition. Furthermore, previous studies have found that topiramate promotes neurological recovery in rats after TBI without affecting the final size of the traumatic lesion and that it might play a role in the reduction of post-traumatic cerebral edema [14]. 

As to address the patient's visual hallucinations, likely due to CBS, he was prevented from undergoing visual overstimulation during his admission. This was achieved by placing him in an isolation room under one-to-one service to address any needs or concerns. Anecdotally, it has been observed that typical and atypical antipsychotics have shown some benefit in individual patients. Nevertheless, there is little evidence to support this. It may not outweigh the risks of side effects or interactions with other medications [15]. We found that treating the patient's acute distress and decreasing visual overstimulation while providing reassurance served in the regression of visual hallucinations.

It is of note that serotonin modulates mood, arousal, emotion, and working memory, among other states or symptomatologies. Therefore, selective serotonin reuptake inhibitors (SSRIs) constitute an attractive, titratable, and potentially long-term pharmacological intervention for post-TBI neurocognitive and neuropsychiatric deficits [16]. In this setting, it was observed that our patient's condition began to improve after the administration of escitalopram in tandem with topiramate.

## Conclusions

It was observed that after stabilizing our patient's acute agitation with sedatives and administering topiramate and escitalopram in tandem with a decrease in visual overstimulation, his altered mental status progressively improved. At the very least, there was a transient regression of his visual hallucinations. Further research must be done regarding the most appropriate treatment for traumatic brain injury sequelae and the relationship between Charles Bonnet syndrome and traumatic brain injury. This syndrome has been observed in blind and non-blind patients after a traumatic brain injury, and its etiology must be further explored. We aim to further medical literature on this subject and continue to raise awareness of some of the rare complications of traumatic brain injury, as described in this case. 
